# Prevalence, characterization, and antibiotic susceptibility of *Cronobacter* spp. in a milk powder processing environment: The first reported case in Serbia

**DOI:** 10.1002/fsn3.2681

**Published:** 2021-12-20

**Authors:** Csaba Csorba, Marija Pajić, Bojan Blagojević, Stephen Forsythe, Miodrag Radinović, Branko Velebit

**Affiliations:** ^1^ Department of Veterinary Medicine Faculty of Agriculture University of Novi Sad Novi Sad Serbia; ^2^ foodmicrobe.com Nottingham UK; ^3^ Department of Microbiology and Molecular Biology Institute of Meat Hygiene and Technology Belgrade Serbia

**Keywords:** antimicrobial resistance, biofilm production, *Cronobacter sakazakii*, MLST, powdered milk, prevalence

## Abstract

*Cronobacter* spp. are opportunistic foodborne pathogens that most often infect neonates and infants through contaminated powdered infant formula. No reports have been published in Serbia on the prevalence of *Cronobacter* spp. in powdered milk production environments. Consequently, this study aimed to determine the prevalence, molecular characterization, antimicrobial susceptibility, and biofilm‐forming ability of *Cronobacter* spp. isolated from a milk powder plant. Hundred samples were collected from the production facility. Fifteen *Cronobacter sakazakii* strains were isolated and identified, giving a contamination rate of 15%. Using multi‐locus sequence typing, the isolates were divided into five sequence types (STs). *Cronobacter sakazakii* ST4 (50%), ST1 (16.67%), and ST83 (16.67%) were the dominant STs isolated. A novel sequence type (ST759) was identified and registered in the *Cronobacter* MLST database. The results of the antibiotic susceptibility testing indicated that *C. sakazakii* strains were susceptible to piperacillin/tazobactam, ampicillin/sulbactam, and amoxicillin/clavulanate, especially to meropenem and cefotaxime. Most of the ST4 showed moderate‐to‐strong biofilm‐forming ability. The presence of clinically relevant isolates (ST4, ST1, ST83, and ST8) revealed that the production plant is likely a potential concern for public health. Finally, finding new sequence types like the one detected in this study (ST759) underlines evolving genetic changes in *C. sakazakii*.

## Graphical abstract

This study aimed to determine the prevalence, molecular characterization, antimicrobial susceptibility, and biofilm‐forming ability of *Cronobacter* spp. isolates from a milk powder plant. Hundred samples were collected from the production facility. A novel sequence type was identified and assigned in the *Cronobacter* MLST database under the ST759 label. The presence of clinically relevant isolates (ST4, ST1, ST83, and ST8) revealed that the production plant is likely to be a potential concern for public health.

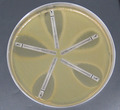

## 1 INTRODUCTION

*Cronobacter* spp. are opportunistic foodborne pathogens, which most often attack prematurely born neonates, causing sepsis, necrotizing enterocolitis, and meningitis, with a mortality rate of 40%–80% (Henry & Fouladkhah, 2019; Holý & Forsythe, 2014). Diseases of infants caused by *Cronobacter* spp. are mainly associated with consuming contaminated powdered infant formula (PIF). These organisms have been isolated from fresh and dried herbs, spices, cheese, cheese products, minced beef, sausages, different vegetables, dry cereals, chocolate, pasta, milk powder, infant weaning formula, and powdered infant formula (Joseph et al., 2012; Kalyantanda et al., 2015; Ueda, 2017). This pathogen has been isolated from various herbal teas in Serbia (Jošić et al., 2017); however, no reports have been published on the incidence rate of *Cronobacter* spp. in the Serbian dairy production plants, especially in powdered milk production environments.

The genus *Cronobacter* consists of seven species: *Cronobacter sakazakii*, *Cronobacter malonaticus*, *Cronobacter universalis*, *Cronobacter turicensis*, *Cronobacter dublinensis*, *Cronobacter condimenti*, and *Cronobacter muytjensii* (Farmer, 2015; Forsythe, 2018). *Cronobacter* spp. are Gram‐negative microorganisms that are motile, peritrichously flagellated, nonsporogenic, nonacid‐fast, straight, and rod‐shaped. Their size varies from 1 µm to 3 µm, but some can be up to 6 µm in size.

*Cronobacter,* in addition to various biotic ones, also survives well on abiotic surfaces, such as stainless steel and polyester plastic. The microorganism can also form an adherent (sessile) community of bacterial biofilms in biotic and abiotic environments. One of the characteristics of the bacterium is the tendency to create a polyanionic extracellular polysaccharide, also known as ESP (Ling et al., 2020). Within the powdered milk plant, layers are forming on surfaces that are in constant contact with nutrients. This phenomenon can lead to accelerated biofilm production of the observed pathogen (Huang et al., 2020).

*Cronobacter* was previously reported to be found in PIF samples and the environment of the powdered milk production plant, with the dominance of *Cronobacter sakazakii* clonal complex 4 (Sonbol et al., 2013). In the powdered infant formula production process, the addition of heat‐sensitive material, spray drying, fluidized bed‐drying, filling, and packing could be the possible links with *C. sakazakii* contamination (Fei et al., 2015; Sonbol et al., 2013). Therefore, the presence of *Cronobacter sakazakii* in powdered milk plants and commercial PIF needs to be monitored.

Antimicrobial susceptibility of *Cronobacter* strains toward the ciprofloxacin, kanamycin, furazolidone, spectinomycin, sulfonamides, gentamicin, cefoxitin, ceftriaxone, and meropenem was demonstrated in several studies (El‐Sharoud et al., 2009; Jaradat et al., 2014; Li et al., 2016). The same studies showed *C. sakazakii* resistance to tetracyclines, ampicillin, neomycin, amoxicillin, chloramphenicol, cephalothin, and rifampicin. Pakbin et al. (2021) reported multidrug resistance to ampicillin, amoxicillin, tetracycline, and ciprofloxacin.

Farhoudi et al. (2019) used real‐time polymerase chain reaction (RT‐PCR) for rapid detection and enumeration of bacterial load in pasteurized milk samples. This method can precisely detect and enumerate bacterial pathogens; nonetheless, it is fast and very accurate. The RT‐PCR method showed a more coherent and focused result, with an approximately overall analyzing period of 3 h compared with conventional culture methods. Several techniques can be used to explore the genetic diversity of *Cronobacter* spp. Pulsed field gel electrophoresis (PFGE), BOX‐polymerase chain reaction (BOX‐PCR), repetitive sequence‐based PCR (rep‐PCR), random amplification of polymorphic DNA (RAPD), enterobacterial repetitive intergenic consensus PCR (ERIC‐PCR), ribotyping methods, and multi‐locus sequence typing were previously used for genotyping of *Cronobacter* (Forsythe, 2018). Pakbin et al. (2021) used the random amplified polymorphic DNA (RAPD) method to investigate genetic relatedness between isolates originated from different sources.

Additionally, to determine phylogenetic relatedness, sequence data from more than one gene can be used to reduce the number of possible doubts caused by genetic recombination or specific selection. Thanks to this, species identification and determination of intra‐ and intergeneric relationships are much more reliable than in 16s rDNA gene sequencing (Baldwin et al., 2009; Dauga, 2002). The work of Baldwin et al. (2009) focused on a higher resolution analysis of *Cronobacter* using seven loci (*atpD*, *fusA*, *glnS*, *gltB*, *gyrB*, *infB*, and *ppsA*). Multi‐locus sequence typing gives a clearer picture of the pathogen itself and potential mutations in the pathogen's genetic material in the form of a different nucleobase arrangement. By identifying *Cronobacter* in a production facility and finding possible mutations using the MLST analysis, a considerable contribution can be made to public health in the form of disease prevention itself.

The main objective of this study was to determine the prevalence of *Cronobacter* spp. in the milk powder production plant located in Serbia, to perform genetic diversity analysis by MLST and to explore the association between biofilm formation and antibiotic resistance among food and environmental *Cronobacter* spp. strains. The primary hypothesis was that *Cronobacter* spp. could be present in milk powder plants in Serbia.

## 2 MATERIALS AND METHODS

### 2.1 Milk powder plant

The dairy factory investigated in this study produced a variety of dairy products including milk powder for sale. The product was partially agglomerated skim milk powder in 25‐kg bags. The product was stored at ambient temperature, preferably lower than 30°C, with relative humidity not higher than 70%. The shelf life of the end product under these storage conditions would be not less than 9 months.

The factory with its 15,700‐m^2^ production area accommodating 40,000 L of milk as daily processing capacity has been operating ever since 1980s and has implemented the ISO 22000, HACCP, and IFS standards. HACCP plan included steps after pasteurized milk storage to product storage and considered health hazards. Potential microbiological hazards were identified in the following process steps: preheat treatment, concentration, balance tank and spray dryer operations, fluid bed dryer, pipelines, cyclones, and packaging. Also, a program of environment control in powder manufacturing, packaging, and surrounding areas has been laid down due to environmental contamination with microorganisms through air and aerosols of powder.

Biological hazards in self‐control plans encompassed testing against *Salmonella* spp., *Listeria monocytogenes*, *Escherichia coli*, *Staphylococcus aureus*, molds, and *Enterobacteriaceae*. There was no process hygiene criterion set for *Cronobacter* spp. Instead, production facility monitored the processing areas and equipment for *Enterobacteriaceae* as part of their sampling plan.

Facility records showed occasional presence of *Enterobacteriaceae* in dried milk, which was mostly attributed to incidental contamination via the large volume of air in drying and cooling operations.

### 2.2 Sample collection

Powdered milk and environmental samples were collected during 2020 from a milk powder plant located in Serbia. Surface swabs (at least 1,000 cm^2^) were taken from various locations with sterile moistened Sponge‐Sticks (Romer Labs, Newark, USA). Samples were taken from the food contact surfaces and equipment 2 h after cleaning and sanitization. After sampling, the Sponge‐Sticks soaked in neutralizing buffer (10 ml) were placed in sterile bags with the location identification label. Immediately after collection, samples were transported to the laboratory in refrigerated coolers and analyzed within the next 3 h.

A total of 100 samples were collected in the study, including 80 swab samples from raw milk bulk storage (20 samples), pasteurizer pipelines (20 samples), evaporator premises (20 samples), spray dryer chamber (20 samples), and 20 samples of dry milk powder from baghouse. The samples were taken in spring (March 2020), summer (July 2020), autumn (October 2020), and winter (December 2020), respectively. During each sampling session, a total of 25 samples were collected, and five samples each from the places mentioned above. The selected sampling sites corresponded to the areas where *Cronobacter* spp. can grow and survive after sanitation procedures, being continuously contaminated, such as on the surfaces of an irregular shape. Also, sampling was done on locations where a transfer of microorganisms from one site to another is probable but is not continuously contaminated. Estimating the number of samples needed to confirm the absence/presence of a *Cronobacter* spp. with a confidence level of 95% and assumed a minimum level of contamination was calculated according to the formula published by Zoellner et al. (2018).

### 2.3 Isolation and identification of *Cronobacter* spp

The presence of *Cronobacter* spp. in powdered milk samples and environmental swabs was determined by the SRPS EN ISO 22964:2017 method. The method consisted of nonselective pre‐enrichment, enrichment in a selective medium, isolation, and identification of *Cronobacter* spp. on chromogenic agar, and confirmation of potential colonies. Nonselective pre‐enrichment was performed by homogenizing a 25‐g powdered milk sample with 225 ml of buffered peptone water (BPW; Oxoid). Each bag containing an environmental sample (sponge) and neutralizer was added 90 ml of BPW, and the content was thoroughly homogenized by mechanically massaging the sponge. Samples were further incubated at 34°C to 38°C for 18 ± 2 h. Subsequently, 0.1 ml of the BPW suspension was transferred to 10 ml of selective broth, based on lauryl sulfate tryptose broth with sodium chloride and vancomycin (Oxoid). The mixture of suspension and the selective broth was incubated at 41.5°C for 24 ± 2 h. After incubation of the broth mentioned above, the suspension was inoculated utilizing a 10‐µL loop to the chromogenic *Cronobacter* isolation (CCI) agar surface. Chromogenic plates were incubated at 41.5°C for 24 ± 2 h. After incubation, chromogenic plates were examined for typical or suspectable *Cronobacter* spp. colonies, blue‐to‐blue‐green color, and 1–3 mm in diameter.

In order to gain well‐isolated colonies for biochemical characterization, selected colonies were streaked onto the surface of a nonselective tryptone soy agar (Oxoid). Biochemical tests included oxidase test, hydrolysis of 4‐nitrophenyl‐α‐d‐glucopyranoside, l‐lysine decarboxylase test, l‐ornithine decarboxylase test, methyl red test, and Voges–Proskauer reaction. Simultaneously, acid production from d‐arabitol, d‐sorbitol, d‐sucrose, and α‐methyl‐d‐glucoside was tested.

Presumptive *Cronobacter* spp. isolates were stored at −80°C in brain heart infusion broth (Oxoid) with 15% glycerol for further confirmation by PCR assay.

### 2.4 DNA extraction

Isolation of genomic DNA and preparation of the same for real‐time polymerase chain reaction (RT‐PCR) analysis and MLST were performed using a QIAquick PCR Purification Kit (Qiagen) and 1.5 ml of overnight culture grown in tryptic soy broth (TSB), according to the manufacturer's instructions.

### 2.5 Confirmation of *Cronobacter sakazakii* by real‐time PCR

The real‐time PCR method was used to confirm the presence of *C. sakazakii* in powdered milk plants in the present study. The following species‐specific primers of the *cgcA* gene described by Hu et al. (2016) were used: *cgcA*‐forward (5′‐GCAGGTGCTGCTGCGAG‐3′), *cgcA*‐reverse (5′‐CGGGTATGACAAAGACAATCTGCG‐3′), and *cgcA* probe (FAM‐TTGATCAGGTCGTCAGAATCTACGGGT‐BHQ1), and they were synthesized by the Microsynth company (Switzerland). The real‐time PCR mix contained 12.5 µl of 2× Brilliant III Ultra‐Fast QPCR Master Mix (Agilent), 5 µl of DNA templates (50 ng/µl), 1 µl (10 µmol/L) of each primer, 0.5 µl (10 µmol/L) of the probe, and 5 µl of double‐distilled water. The AriaMx real‐time PCR System (Agilent) was used for thermocycling and to measure changes in fluorescence. The PCR was initiated by denaturation at 95°C for 3 min, followed by 40 cycles of denaturation at 95°C for 15 s and annealing at 60°C for 60 s. Fluorescence was measured at 60°C negative controls were included, containing all the elements of the reaction mixture except the template. All samples were processed in triplicate. DNA extracted from the pure culture of *C. sakazakii* ATCC 29544 was used as a positive control.

### 2.6 Multi‐locus sequence typing (MLST) and sequence analysis

The MLST gene set was amplified using primers and PCR conditions according to protocol available at the MLST *Cronobacter* database (https://pubmlst.org/organisms/cronobacter‐spp; Baldwin et al., 2009). The gene loci used in this study were as follows: *atpD*, *fusA*, *glnS*, *gltB*, *gyrB*, *infB,* and *ppsA*.

Reaction conditions for all the primers were as follows: initial denaturation at 94°C for 2 min, 30 cycles of denaturation at 94°C for 1 min, primer annealing at 58°C for 1 min, and extension at 72°C for 2 min, followed by a final extension step at 72°C for 5 min. Each 50‐µl amplification reaction mixture contained 20 ng chromosomal DNA, 10 µl Q solution (Qiagen), 20 pmol forward and reverse primer, and 1 × PCR buffer (Qiagen), which consisted of 1.5 mM MgCl_2_, 0.8 mM deoxynucleotide triphosphates, and 1.25 U Taq (Qiagen). According to the manufacturer's protocol, each amplicon was purified before sequencing with MinElute UF microtiter plates (Qiagen).

In multi‐locus sequence typing, nucleotide sequences were determined using nested primers for sequencing at least once on each DNA strand using a BigDye Terminator Ready Reaction Mix v3.1 (PE Biosystems) under standard sequencing conditions, according to the manufacturer's protocol. Unincorporated dye terminators were removed by precipitation with 95% alcohol. Reaction products were separated and detected on an ABI PRISM 3100 genetic analyzer (PE Biosystems), using a standard sequencing module with a Performance Optimized Polymer and 5‐cm array. Sequences originating from both strands of the observed locus of the same isolate were aligned, trimmed to the desired length, and edited using the SeqMan II software (DNA Star Software, Madison, USA).

Arbitrary allelic numbers were assigned to each unique allele for the observed locus according to the PubMLST (public databases for molecular typing and microbial genome diversity) scheme. After sequencing and assigning allele types to each of the selected seven loci, the allelic profile was created by combining seven numbers, representing the sequence type (ST) for that particular isolate (e.g., ST7). A novel sequence type (ST) designation was assigned by the database curator to each isolate with a unique allelic profile. In contrast, subsequent isolates with an identical allelic profile were classified into the same sequence type identifier. They considered isogenic strains because they did not show a noticeable difference at all seven loci. All alleles within the MLST scheme were located within the framework and served as references for analysis.

Linkage analysis was performed using the index of association (*I_A_*). It was examined whether the alleles were randomly linked; that is, the mentioned linkage was in equilibrium, which would indicate a freely recombinant population. When the association is in equilibrium, or in other words, when there is a random association between alleles of different loci present, the association index is equal to zero (*I_A_* = 0). Suppose the value of *I_A_* differs significantly from zero, then it indicates rarely present or completely absent recombination within the population, in which case we are talking about the clonal structure of the population.

The nucleotide sequences of each gene were trimmed to the appropriate length and then queried to the MLST database. Finally, allele numbers were assigned, and STs were determined by the tools available at the MLST *Cronobacter* database. The designation of new alleles and STs was determined by the MLST database curator (SJF).

### 2.7 Antimicrobial susceptibility testing

The Epsilometer test (Etest) method was chosen based on the conclusions of Ogata et al. (2014), who showed that the Etest was the best option to routinely perform susceptibility testing, especially to tetracycline, followed by amoxicillin, clarithromycin, and metronidazole. Besides, the Etest method presented a better agreement with the agar dilution method. The disk‐diffusion method pointed out a high disagreement with the agar dilution method and an excessive rate of major errors. Minimum inhibitory concentrations (MICs) for the *Cronobacter sakazakii* isolates were determined by the Etest (bioMérieux) on Mueller‐Hinton agar (MHA; Oxoid), according to the manufacturer's instructions. The following antibiotics were tested: piperacillin/tazobactam (β‐lactam/β‐lactamase inhibitor), ampicillin/sulbactam (β‐lactam/β‐lactamase inhibitor), amoxicillin/clavulanic acid (β‐lactam/β‐lactamase inhibitor), meropenem (carbapenem), and cefotaxime (cephalosporin/cephamycin). Summarily, suspensions of an overnight culture of the isolates were prepared in sterile phosphate‐buffered saline (Merck Millipore) to a density that matched a 0.5 McFarland turbidity standard. These inoculums were evenly spread on the surface of MHA using a cotton swab and allowed to dry for at least 10 min before the five Etest strips were radially applied onto the surface of a 140 × 20 mm Petri dish (Nunc), and duplicate tests were performed. The plates were incubated at 35 ± 2°C for 16–20 h in an aerobic incubator.

**FIGURE 1:**
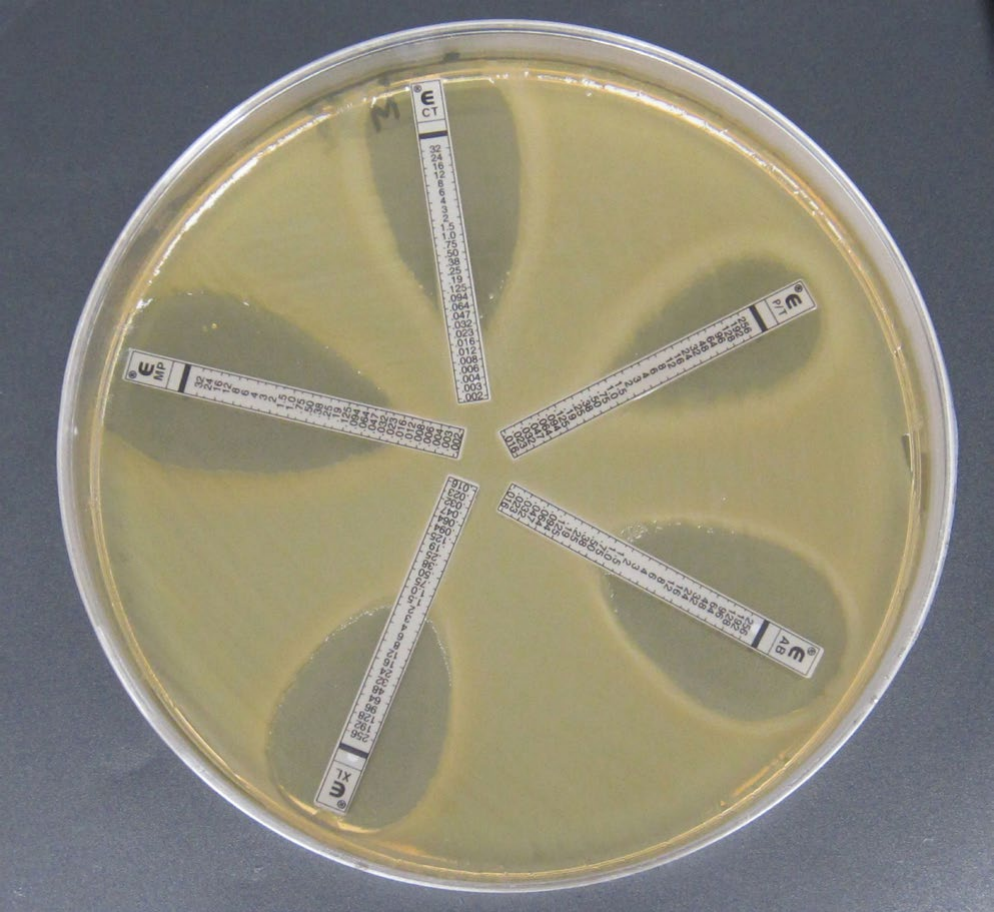
Etest MICs of piperacillin/tazobactam (P/T), ampicillin/sulbactam (AB), amoxicillin/clavulanic acid (XL), meropenem (MP), and cefotaxime (CT) for an environmental isolate of *Cronobacter sakazakii*

The MICs were interpreted by reading the intercept of the inhibition zone and the Etest strip (Figure 1). A higher MIC was selected when replicates generated different MIC values. *Escherichia coli* ATCC 25922 was used as the quality control organism.

### 2.8 Biofilm‐forming ability

The biofilm‐forming ability of *Cronobacter sakazakii* was tested by an indirect quantitative method, the so‐called crystal violet method, using microtiter plates. In the performance of the crystal violet method, 96‐well, flat‐bottomed, polystyrene microplate (Nunc) was used, in which suspensions of *C. sakazakii* isolates were placed. For each isolate, three wells on a microplate were inoculated with 180 µl of TSA broth, after which 20 µl of overnight suspension of the corresponding isolates was added. The negative control contained only the stated amount of 200 µl. The plates were incubated for 48 h at 25°C. After the incubation, nonadherent cells were removed by inverting the microplate on an absorbent medium. The rest of the nonadherent cells and the excess medium were removed by rinsing three times with 250 µl of sterile saline solution. Bound *C. sakazakii* bacteria were fixed by drying inverted at room temperature. After drying, 250 µl of 0.5% crystal violet solution (Merck Millipore) was added to each well, after which the microplate was incubated for 10 min at room temperature. Excess unbound dye was removed by repeated rinsing with sterile saline solution until the dye residue was removed entirely. In the next step, the microplate was dried inverted at room temperature. The crystal violet, which remained bound to the adherent bacteria, was dissolved by adding a decolorizer (a mixture of ethanol and acetone in a ratio of 80:20) in an amount of 250 µl. The plate was left for 15 min at room temperature to achieve complete decomposition of the dye.

The results were read using a spectrophotometer at 595 nm (Eppendorf). The absorbance values used to characterize the biofilm‐forming ability of the tested isolates were obtained by reducing each absorbance by the mean absorbance value of pure broth (negative control). Isolates were classified into four categories according to Stepanović et al. (2007).

## 3 RESULTS

### 3.1 Isolation, identification, and prevalence of *Cronobacter* spp

*Cronobacter* spp. were isolated from 15 out of 100 (15%) samples collected from a powdered milk plant. All isolates from the samples formed small convex blue‐to‐blue‐green colonies on CCI agar plates. These colonies were considered as *Cronobacter* spp. in this research. The biochemical characterization tests of 15 *Cronobacter* isolates demonstrated the following reactions: negative oxidase test, a positive test for hydrolysis of 4‐nitrophenyl‐α‐d‐glucopyranoside, a negative test for l‐Lysine decarboxylase and l‐Ornithine decarboxylase, acid from d‐sucrose and a methyl‐α‐d‐glucoside, and no acid from d‐arabitol and d‐sorbitol. All 15 isolates were confirmed as C. *sakazakii* by the real‐time PCR method.

The highest percentage of C. *sakazakii* isolates were found in July (33.33%, 5 out of 15) and October (33.33%, 5 out of 15), followed by December (20%, 3 out of 15) and March (13.33%, 2 out of 15). In the case of sampling sites, the highest number of C. *sakazakii* positive samples was identified in dry powder from baghouse (46.67%, 7 out of 15), followed by swabs collected from spray dryer chamber (40%, 6 out of 15), and environmental swabs collected in evaporator premises (13.33%, 2 out of 15).

### 3.2 MLST analysis of *Cronobacter sakazakii* isolated from the powdered milk plant

Using 7‐locus MLST, the 15 *C. sakazakii* strains were analyzed. The analysis on the three of the tested strains (2 strains from dry powder from baghouse, one strain from environmental swabs from evaporator premises) was found difficult due to their weak signal. Hence, they were dropped from the study. After the MLST analysis, the 12 *C*. *sakazakii* strains were clustered into five sequence types, shown in Table 1.

**TABLE 1:** Results of MLST analysis of *Cronobacter sakazakii* strains isolated from powdered milk plant in Serbia

PubMLST strain identification code (ID)	Isolate identification code	Source of the strain	The month of sample collection	Sample type	*atpD*	*fusA*	*glnS*	*gltB*	*gyrB*	*infB*	*ppsA*	Sequence type (ST)	Clonal complex
3182	M46	Powdered milk	6	End of production	5	1	3	3	5	5	4	4	4
3183	M71	Powdered milk	10	End of production	11	8	7	5	8	15	10	8	8
3184	M72	Powdered milk	10	End of production	5	1	3	3	5	5	4	4	4
3185	M97	Powdered milk	12	End of production	19	16	19	41	19	15	23	83	83
3186	M98	Powdered milk	12	End of production	5	1	3	3	5	5	4	4	4
3187	S38	Environmental	6	Evaporator premises	5	1	3	3	5	5	4	4	4
3188	S43	Environmental	6	Swab from spray dryer	5	1	3	3	5	5	4	4	4
3189	S45	Environmental	6	Swab from spray dryer	5	1	3	3	5	5	4	4	4
3190	S66	Environmental	10	Swab from spray dryer	1	1	1	1	1	1	1	1	1
3191	S70	Environmental	10	Swab from spray dryer	1	1	1	1	1	1	1	1	1
3192	S93	Environmental	12	Swab from spray dryer	19	16	19	41	19	15	23	83	83
3193	S16	Environmental	3	Swab from spray dryer	15	205	13	94	99	118	126	759	

All sequence types were in the species *Cronobacter sakazakii*. It means that *C. sakazakii* was the only dominant species isolated from dry powder and its production environment. The main *Cronobacter sakazakii* sequence types were *C. sakazakii* ST4 (50%, 6 out of 12), ST1 (16.67%, 2 out of 12), ST83 (16.67%, 2 out of 12), ST8 (8.33%, 1 out of 12), and a novel sequence type 759 (8.33%, 1 out of 12). The only isolated sequence type from environmental swabs collected in evaporator premises was ST4. The situation regarding the sequence type distribution was equal in swabs collected from the spray dryer chamber with ST4 (2/6) and ST1 (2/6). Unlikely, ST4 (3/5) was dominant in dry powder collected from the baghouse. One of the five sequence types isolated had not been reported before and consequently was assigned as a new sequence type in the *Cronobacter* MLST database under the ST759 label. The new allele numbers assigned were as follows: *atpD* 15, *fusA* 205, *glnS* 13, *gltB* 94, *gyrB* 99, *infB* 118, and *pps* 126.

### 3.3 Antimicrobial susceptibility

The minimal inhibitory concentration (MIC) of the 15 *Cronobacter* strains is shown in Table 2. Antibiotic susceptibility profiling indicated that all 15 isolates were sensitive to all antimicrobial agents investigated. No drug or multiple drug resistance was detected.

**TABLE 2:** Minimal inhibitory concentration (MIC) values and sequence types (STs) of *Cronobacter sakazakii* strains isolated from processing environments and PIF with cutoff values for *Enterobacteriaceae*

Isolate identification code	Sequence type (ST)	MIC (µg/ml)				
		Antibiotics				
		Piperacillin/Tazobactam	Ampicillin/Sulbactam	Amoxicillin/Clavulanate	Meropenem	Cefotaxime
S38	ST4	2	1.5	4	0.094	0.125
S43	ST4	4	2	4	0.032	0.25
S45	ST4	4	1.5	5	0.032	0.125
M46	ST4	3	3	6	0.032	0.19
M72	ST4	4	4	8	0.047	0.25
M98	ST4	2	2	4	0.032	0.094
S66	ST1	2	2	4	0.032	0.125
S70	ST1	4	2	6	0.064	0.125
S93	ST83	1	1.5	2	0.016	0.094
M97	ST83	2	0.75	3	0.023	0.094
M71	ST8	0.75	0.75	2	0.016	0.032
S16	ST759	2	2	3	0.023	0.125
M22	–	2	1.5	3	0.032	0.064
M49	–	3	1	3	0.047	0.064
S63	–	3	3	4	0.032	0.19
Cutoff value for *Enterobacteriaceae* by EUCAST (2021)		8	8	8	2	1

### 3.4 Biofilm‐forming ability

The microtiter plate assay detected the biofilm‐forming ability among the 15 *C. sakazakii* isolates. The cutoff OD (ODc) was defined as three standard deviations (SD) above the mean OD (optical density) of the negative controls. Based on the ODc, the *C. sakazakii* isolates were classified into four categories: OD of the test isolate ≤ ODc (0.212)—no biofilm production, ODc (0.212) ≤ OD of the test isolate ≤ (2 × ODc (0.424))—weak biofilm production, (2 × ODc (0.424)) < OD of test isolate ≤ (4 × ODc (0.848))—moderate biofilm production, and OD of test isolate > (4 × ODc (0.848))—strong biofilm production. The results indicated that all 15 of the tested isolates were capable of producing biofilm on polystyrene microplates. According to the results, one of the two isolates with strong biofilm‐forming ability belonged to ST4 (OD = 1.243). The other one with the strong biofilm‐forming ability (OD = 1.299) was given a low signal during MLST analysis, and it was left out of sequencing. Five out of 15 tested isolates manifested moderate biofilm‐forming ability. Four out of 5 isolates with moderate biofilm‐forming ability belonged to ST4, and the other was identified as ST1. The remaining eight isolates were classified as weak biofilm producers. The distribution of the sequence types with weak biofilm‐forming ability was as follows: ST83 (25%, 2/8), ST4 (12.5%, 1/8), ST1 (12.5, 1/8), ST8 (12.5, 1/8), and ST759 (12.5%, 1/8), including two isolates, which were not classified into sequence types, for the same reason as the previous one.

## 4 DISCUSSION

In Serbia, Jošić et al. (2017) analyzed 360 commercial powdered infant formula samples, but *C. sakazakii* was not detected in these samples. In this research, *Cronobacter sakazakii* was isolated from a powdered milk plant, and the prevalence of the pathogen was 15%. In comparison with the results of Fei, Jiang, Jiang, et al. (2017), who described *C. sakazakii* prevalence ratio of 5.7% in commercial PIF, the results of this research show a higher prevalence rate. Also, Fei et al. (2015) reported a 7.5% prevalence of positive *Cronobacter* strains isolated from an infant formula production factory in China, which is lower than the results of this research. First of all, the current prevalence ratio of isolated *Cronobacter* strains from a powdered milk plant could be the consequence of noncompliance with the production facility's HACCP plan and general hygiene guidelines. The highest contamination rate was reported from the dry powder collected from the baghouse. This fact can be related to the possible contamination of the final product by the staff's negligence (Gan et al., 2021). Mullane et al. (2008) reported that 100% of collected samples from the filters of the spray dryer were positive for *C. sakazakii* and *C. malonaticus*. Additionally, three *Cronobacter* isolates were isolated from rennet casein powder at a frequency of 25%. In the observed powdered milk plant, the presence of *C. sakazakii* in dry powder from the baghouse was 35% (7 out of 20), followed by swabs collected from spray dryer chamber with 30% (6 out of 20), and environmental swabs collected in evaporator premises with 10% (2 out of 20). The facts mentioned above indicate that air circulation between the spray dryer facility and the baghouse could be the main route for product contamination with *C. sakazakii*. So, that could reveal the transmission of the pathogen by air in the production plant.

Baldwin et al. (2009) reported the dominant presence of ST4 (36.67%, 22/60) among the tested isolates. They also isolated ST8 (13.33%, 8/60) and ST12, predominantly originating from clinical specimens. In this research, the level of occurrence for ST4 was higher (50%, 6/12), while the level for ST8 (8.33, 1/12) was lower. Sequence type 4 was isolated from environmental swabs collected in evaporator premises, swabs collected from the spray dryer chamber, and dry powder from the baghouse. It is interesting in this connection for us to note that Joseph and Forsythe (2012) found that in cases of neonatal infection, almost half (48.78%, 20 out of 41) of the isolated strains belonged to ST4, followed by ST8 (17.07%, 7 out of 41), and ST1 (9.76%, 4 out of 41). Joseph and Forsythe (2012) later revealed the high clonality of ST4 and association of clonal complex 4 with cases of neonatal meningitis. From the above, the predominance of ST4, particularly in baghouse, indicates a serious concern in terms of public health. Hariri et al. (2013) stated that *C. sakazakii* ST4 causes severe meningitis in infants. They only isolated ST4 from cerebrospinal fluid (CSF). The dominant sequence type in commercial PIF and infants exposed to PIF was ST8, who had diarrheal symptoms. The appearance of ST4 and ST8 in baghouse can be a serious concern because meningitis associated with diarrhea can lead to fatal outcomes in preterm infants and neonates. The original study by Sonbol et al. (2013) reported that 21 out of 72 *C. sakazakii* strains were in the clinically significant ST4 clonal complex. They described that ST4 and ST1 are not closely related, and ST1 was primarily isolated from powdered milk plants or powdered infant formula production environments. In this research, ST1 (16.67%, 2 out of 12) was only isolated from the spray drier chamber, but as described by Sonbol et al. (2013), in most cases, ST1 appears together with ST4. Chase et al. (2017) tested the potential in vivo pathogenicity of the ST83 strains. The results showed a high mortality rate (90%–100%) for all of these strains, suggesting a very high risk for neonates exposed to PIF contaminated with *Cronobacter sakazakii* ST83. They described that ST83 could persist within a PIF manufacturing facility and potentially set up significant quality assurance challenges to the manufacturing industry. Forsythe et al.'s (2014) study marked out that *C. sakazakii* ST83 especially caused septicemia in infants and was mainly isolated from environmental and infant formula samples. In this study, ST83 was found in swabs from the spray dryer chamber and dry powder from the baghouse. This fact is worrying, considering that ST4 and ST1 are present in equal proportions in the dryer chamber, which we know to be very dangerous. While in the packing room, the mentioned sequence type is together with the highly pathogenic ST4. The isolated novel sequence type had a unique gene locus structure. The mentioned sequence type should be further investigated. The results of this research correlate with the results of Fei et al. (2015), Ogrodzki and Forsythe (2017), and Fei, Jiang, Jiang, et al. (2017).

Antimicrobial susceptibility testing was performed in the case of five most commonly used antimicrobial drugs in the therapy of infants. It is widely known that the excessive use of antibiotics in veterinary medicine and human healthcare facilities increased the occurrence level of resistant bacterial strains. In terms of *Cronobacter* spp., no official clinical breakpoints were established by EUCAST neither CLSI for the 2021 year. The mentioned breakpoints were only present for *Enterobacteriaceae*, which are not satisfactory for an accurate assessment of *Cronobacter* spp. antibiotic susceptibility. In this study, EUCAST cutoff values for *Enterobacteriaceae* were used to classify the antimicrobial susceptibility. The results shown in Table 2 were similar to the findings of Brandão et al. (2017) and Holý et al. (2019).

Furthermore, we found that the MIC values of piperacillin/tazobactam, ampicillin/sulbactam, and amoxicillin/clavulanate varied within each ST and across different STs. These findings partially agree with Fei, Jiang, Feng, et al.'s (2017) reports, who found that MIC values were similar within STs. A possible explanation would be the overuse of piperacillin/tazobactam, ampicillin/sulbactam, and amoxicillin/clavulanate in veterinary practice, as was demonstrated by Zarzecka et al. (2021). Pakbin et al. (2021) reported high resistance levels to ampicillin, amoxicillin, and chloramphenicol. Furthermore, they revealed 6 *C. sakazakii* isolates with multidrug resistance to amoxicillin and ampicillin. In this research, multidrug‐resistant isolates were not discovered. When it comes to meropenem and cefotaxime, respective MIC values were almost similar within the same ST. This fact indicates that a connection between the ST and antimicrobial susceptibility may be present. The results of this research are completely correlating with the results of Fei, Jiang, Jiang, et al. (2017). They reported that all 56 *C. sakazakii* strains isolated from commercial PIF were susceptible to ampicillin‐sulbactam, cefotaxime, meropenem, and piperacillin‐tazobactam.

Similarly, Brandão et al. (2017) revealed that *Cronobacter* spp. strains isolated from food samples were susceptible to all the antibiotics tested. Mardaneh and Soltan Dallal (2017) described that one *C. sakazakii* strain originating from PIF was resistant to meropenem. However, no resistance toward meropenem was revealed in this research. According to the study of Parra‐Flores et al. (2018), the isolated *C. sakazakii* strains were resistant to the first and second cephalosporin generations. Still, they were sensitive to cefotaxime (third cephalosporin generation) and cefepime (fourth cephalosporin generation). The results obtained in this study were comparable with the results mentioned above, that is, showing the lowest MIC values for meropenem and cefotaxime. These findings might occur due to the less frequent use of these antibiotics in dairy cow therapy compared to the use of β‐lactams and β‐lactamase inhibitors (Darko et al., 2017; Zhou et al., 2015). Another good sign could be that MIC values for meropenem and cefotaxime vary in a narrow range, in the case of ST4, ST1, and ST83, which were described as the most pathogenic sequence types. The novel sequence type ST759 showed a high susceptibility rate toward all the tested antibiotics.

Li et al. (2017) reported that 13 (32.5%) of the isolated *Cronobacter* strains from spices and cereals were classified as weak biofilm producers, one (2.5%) was a moderate biofilm producer, and one (2.5%) was strong biofilm producer. These authors stated that there was no correlation between biofilm formation and sequence types. In this study, 2 (13.33%) isolates were marked as strong biofilm producers, 5 (33.33%) strains were recognized as moderate biofilm producers, and 8 (53.33%) isolates were categorized as weak biofilm producers. One of the ST4 isolates (M72) showed a strong biofilm‐forming ability and the highest MIC values for all the tested antibiotics. This finding could mean that the higher biofilm‐forming ability of the bacteria plausibly correlates with increased antimicrobial resistance (Neopane et al., 2018). Most of the ST4 showed a moderate‐to‐strong biofilm‐forming ability, especially in dry powder collected from the baghouse, indicating that the mentioned point is critical in the specific production plant regarding public health risk. The results of Du et al. (2018) are in correlation with the results of this research. The novel ST759 showed a weak biofilm‐producing ability, which, in light of high sensitivity to all the tested antibiotics, may be a favorable sign from the aspect of public health.

## 5 CONCLUSIONS

The present research demonstrated that the prevalence of *C. sakazakii* in milk powder facility is 15%. Real‐time PCR analysis revealed that *C. sakazakii* was the only species recovered from environmental swabs and from the dry powder in baghouse. MLST analysis showed a moderate‐to‐high genetic diversity of isolated *C*. *sakazakii* strains. The presence of isolates of clinical relevance, such as ST4, ST1, ST83, and ST8, indicated that the production plant could likely be the potential source of contamination for PIF ingredients. It is encouraging that all isolates were susceptible to antibiotics usually prescribed for children, especially to meropenem and cefotaxime. One of the ST4 isolates (M72) showed a strong biofilm‐forming ability and the highest MIC values for all tested antibiotics. This finding could indicate association between higher biofilm‐forming ability and increased antimicrobial resistance. Most of the ST4 showed a moderate‐to‐strong biofilm‐forming ability, especially in dry powder collected from the baghouse, which indicates this point is critical in evaluation of existing HACCP self‐control plans.

In this study, a novel sequence type with a unique gene locus structure was isolated (ST759), which requires further investigation to reveal its potential pathogenic potential. This finding underlines evolving genetic changes in *C. sakazakii*. Whether this is just a static episode or continuous evolution governed by the paragenetic factors present in the milk powder production environment or not remains to be explored yet.

## Data Availability

The data that support the findings of this study are available from the corresponding author upon reasonable request.
